# Aortic valve calcification in the era of non-coding RNAs: The revolution to come in aortic stenosis management?

**DOI:** 10.1016/j.ncrna.2020.02.005

**Published:** 2020-02-29

**Authors:** Joseph Nader, Laurent Metzinger, Pierre Maitrias, Thierry Caus, Valérie Metzinger-Le Meuth

**Affiliations:** a Department of Cardiac Surgery, Amiens University Hospital, Amiens, France; b HEMATIM EA4666, C.U.R.S, Université de Picardie Jules Verne, 80025, AMIENS Cedex 1, France; c Department of Vascular Surgery, Polyclinique Saint Côme, Compiègne, France; d INSERM U1148, Laboratory for Vascular Translational Science (LVTS), UFR SMBH, Université Paris 13-Sorbonne Paris Cité, 93017, BOBIGNY CEDEX, France

**Keywords:** Aortic stenosis, microRNA, Translational research, Biomarker

## Abstract

Aortic valve stenosis remains the most frequent structural heart disease, especially in the elderly. During the last decade, we noticed an important consideration and a huge number of publications related to the medical and surgical treatment of this disease. However, the molecular aspect of this degenerative issue has also been more widely studied recently. As evidenced in oncologic but also cardiac research fields, the emergence of microRNAs in the molecular screening and follow-up makes them potential biomarkers in the future, for the diagnosis, follow-up and treatment of aortic stenosis. Herein, we present a review on the implication of microRNAs in the aortic valve disease management. After listing and describing the main miRNAs of interest in the field, we provide an outline to develop miRNAs as innovative biomarkers and innovative therapeutic strategies, and describe a groundbreaking pre-clinical study using inhibitors of miR-34a in a pre-clinical model of aortic valve stenosis.

## 1 Introduction

During the last decade, the management of aortic valve stenosis was one of the most mediatized items in cardiovascular medical and surgical meetings around the world. The emergence of transcatheter therapies [1], first in inoperable and high-risk patients in the mid 2000's, has changed the medical practice and widened their indications to become acceptable even in patients with low surgical risk [2, 3]. Meanwhile, the calcified aortic valve has been analyzed more closely, using novel imaging techniques such as enhanced CT scan analysis of the shape, size or calcifications degree of this valve, or with the more recent echocardiographical images in 2D and 3D modeling. However, researchers started to be more interested in the molecular aspect of the calcification process [4], looking for a specific biomarker able to predict calcification progression or severity, in order to prevent it or to improve the minimally invasive follow-up. These molecular interests were accelerated and widened with the emerging of the knowledges on the effect of non-coding RNA in the regulation of cellular and molecular processes, implicated in a large number of diseases.

We report herein a general review on the recent advances in the clinical and molecular aortic stenosis management according to the recent literature.

## 2 Clinical aspect of aortic stenosis

The aortic valve is a part of the aortic root, the door from which the oxygenated blood leaves the left ventricle to the systemic circulation. It is normally constituted from 3 thin leaflets, largely opened in order to evacuate the blood through a 3–4 cm^2^ orifice area. Bicuspid aortic valve occurs in 0.5–1% of the general population and is the most frequent congenital anomaly [5]. In these patients, the aortic valve is composed of only two leaflets, narrowing the aortic orifice causing hemodynamical modifications. These valves degenerate earlier most frequently, on a stenotic pattern, but also by causing aortic regurgitation.

Aortic Stenosis (AS) remains, nowadays, the most frequent cardiovascular disease in the elderly. It occurs in 2–9% of patients older than 65 years old [6,7], with an earlier onset in patients presenting bicuspid aortic valves. The main cause of this stenosis is a fibrous and calcific degeneration of the aortic valve leaflets, occurring on a variable lapse of time, leading to the loss of leaflet plasticity and its rigidification, narrowing the aortic valve orifice. This results in a decrease of the ejected blood volume from the ventricle to the body, leading to the installation of clinical symptoms of aortic stenosis, such as cardiac murmur, dyspnea, angina or even syncope (8). This ventricular outflow obstruction is responsible for an increased ventricular mass and ventricular hypertrophy, resulting in the onset of early signs of left ventricular failure. The severity of aortic valve stenosis is evaluated by echographical evaluation associated to a Doppler analysis of the blood flow across the valve (Table 1) and is classified from mild to severe according to the values of aortic valve area, transvalvular gradients and transvalvular velocity. Aortic valve stenosis can remain asymptomatic until the onset of the first symptoms, many years after the early cellular modifications. At this stage, the prognosis of this disease becomes serious with a rapid drop in the survival, if not treated, with a mortality of nearly 80% at 2 years, especially if LV complications are settled [9].

**Table 1:** Echographical classification of the severity of aortic valve stenosis.

Echographical criteria	Severity of Aortic Valve Stenosis		
	Mild	Moderate	Severe
Effective Aortic Valve Area (cm^2^)	>1,5	1,0–1,5	<1,0
Indexed Effective Aortic Valve Area (cm^2^/m^2^)			<0,6
Mean Transvalvular Gradient (mmHg)	<25	25–40	>40
Transvalvular velocity (m/s)	<3,0	3,0–4,0	>4,0

Surgery remains the gold-standard treatment with an aortic valve replacement under cardiopulmonary bypass, using either mechanical or biological prostheses, after the resection of the native calcified valve. It is a routine intervention, with low perioperative risks when it is not associated to other cardiac or general comorbidities. As we reported earlier, transcatheter therapy emerged since 2002, and became widely used since 2008, with positive results in inoperable and high-risk patients first, then indications became larger to target patients with lower surgical risks as reported in the last randomized studies [10,11]. The choice of the best suited treatment remains a multidisciplinary decision, in the HeartTeam group in each center, in order to propose the most accurate treatment to each patient's situation, according to the latest guidelines [8,12], best benefit/risk ratio, but also, according to the post-operative estimated survival of each patient.

However, and apart from the echographical follow-up of the aortic valve there are no specific, objective and easily measurable marker to analyze the progression of the aortic stenosis. By looking more closely to the molecular and cellular evolution of the aortic valve architecture, can we find something interesting to explore in that way?

## 3 Cellular and molecular aspect of aortic stenosis

**Fig. 1:**
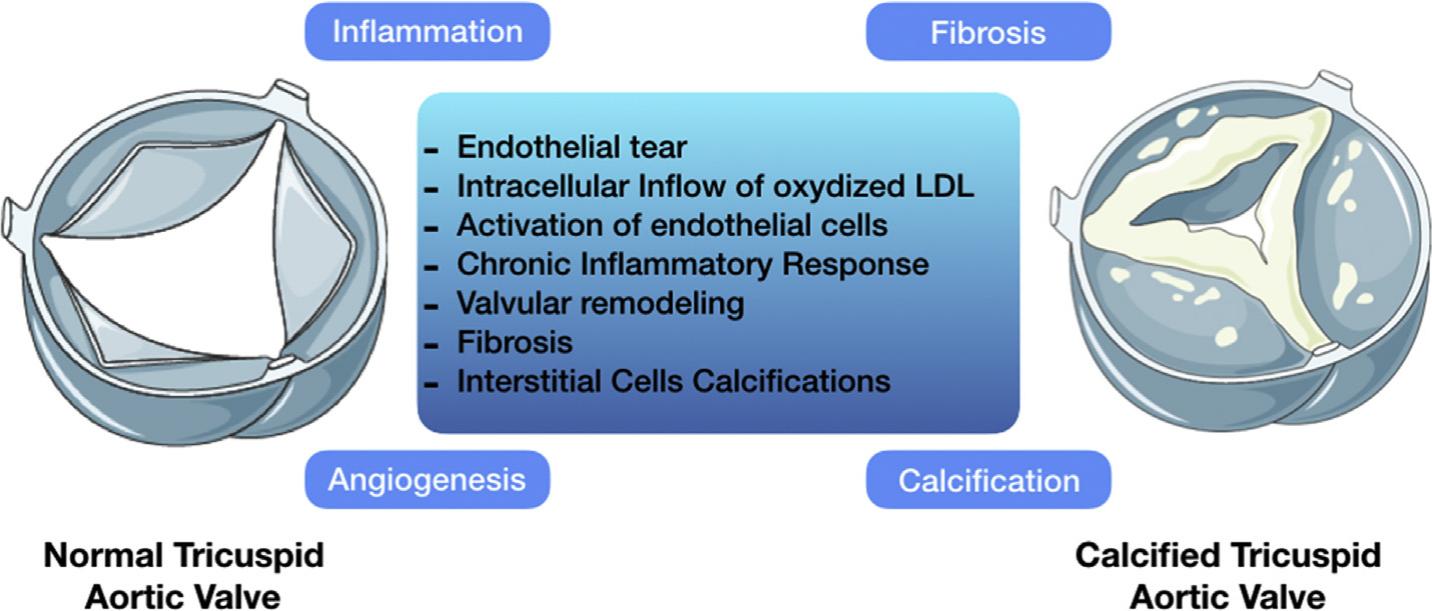
Molecular aspects of aortic valve calcification, showing the major main process implicated in the aortic valve calcifications.

As we mentioned earlier, the aortic valve calcification is a chronic and multifactorial process, starting many years before the onset of clinical symptoms of aortic stenosis [4] with an atherosclerotic-like evolution: inflammation, fibrosis, calcification and neo-angiogenesis [13]. So, it does not result simply from an age-induced degeneration. Multiple factors are implicated in the first step of this degeneration, such as hypertension, dyslipidemia, diabetes, tabagism, stress but mechanical aggression of the endothelial tissue remains the first step in the calcification process. The initial tear in the endothelium or in the ventricularis (ventricular side of the valve) is responsible of the migration of oxidized-LDL molecules in the thickness of the valve [14]. Endothelial cells are activated and an increased expression in e-selectin (*Endothelial Selectin*) and VCAM-1 (*Vascular Celle Adhesion Molecule 1*) [15], enhances the inflammatory response involving T-cells and macrophages, associated with a massive accumulation of extracellular calcium in the inner part of the valve, the fibrosa. This chronic inflammation relaxes inflammation factors such as Tumor Necrosis Factor α (TNF-α), Tumor Growth Factor β (TGF-β) [16], C reactive protein and Interleukin 1β (IL-1β) [17,18], leading to a structural valvular remodeling favoring valvular fibrosis and valvular interstitial cells calcifications. The activated VICs (Valvular Interstitial Cells) over express pro-angiogenic factors such as VEGF [19] and inhibit other anti-angiogenic factors [20,21]. Furthermore, fibrosis and the tissular remodeling enhance the neo-angiogenesis through the TGFβ/Smad pathway [22,23]. The resultant aortic valve calcification has similarities with bone constitution of calcium and hydroxyapatite [24]. These calcifications are the result of the accumulation of oxidized LDL and inflammatory cells [25,26] and may be induced by cellular apoptosis [27,28]. Another hypothesis is that these calcifications follow the pathway of the transformation of the valvular fibroblasts into osteoblasts, secreting procalcifying agents such as ALKL (Alkaline Phosphatase), Bone Morphogenic Protein-2 (BMP-2) and Osteocalcin [4,25,[29], [30], [31], [32]]. BMP initiates the RUNX2/NOTCH1 pathway inducing the osteoblastic differentiation. This pathway expression is modified with the microARN-30b [33] (Fig. 1).

The comprehensive analysis of these complex molecular alterations in the aortic valve induced deep thoughts on the implication of different factors in this mechanism and their potential measure in order to have a clearer idea on the evolution of AS and try to predict its evolution. Which markers have been detected in this disease till now?

## 4 Current biomarkers in aortic stenosis

According to the most recent publications, we did not find any specific marker for the calcifications of the aortic valve. The main biomarkers published and related to AS were mostly indicators of the complications of aortic stenosis, and directly related to the myocardial tissue and global ventricular function.

According to that, we report here a quick summary of these findings (Table 2):

- B-type Natriuretic Peptide and its inactive precursor (NT-pro-BNP) are the most cited predictor factors of AS severity but reflects actually the ventricular consequences of this stenosis, as these myocardial hormones are secreted in case of myocardial tissue dilation [[34], [35], [36], [37], [38], [39]].
- Cardiac Troponin level, also secreted by the myocardial tissue, reflects the muscular ischemic injury such as in myocardial infarction. In AS, the seric detection of high-sensitive troponin is the result of the myocardial fibrosis and ventricular dysfunction, or ischemic attempt of the myocardium because of the decreased coronary flow related to the valvular narrowing. It is then a marker of the advanced aortic stenosis [40].
- Soluble ST2 (sST2) marker is also used in AS. It is implicated in the myocardial remodeling, maybe then in the earliest onset of myocardial modifications secondary to the stenosis [41,42].
- And finally, Galectin-3 is a controversial biomarker of AS because of its implication in the tissular remodeling, myocardial fibrosis and cardiovascular risk [[43], [44], [45], [46]].

**Table 2:** Biomarkers issued from main studies, in relation with the diagnosis and prognosis of aortic stenosis (BNP: B-type Natriuretic Peptide; NT-proBNP; AS: Aortic stenosis; AVR: Aortic Valve Replacement; sST2: soluble ST2; MMP: Matrix Metalloproteases; CV: Cardiovascular).

Biomarker	Role	Author	Sample Size	Results	Ref.
BNP/NT-proBNP	Myocardial Hormone released in response to intracardiac pressure increase	Berger-Klein, 2004	87 symptomatic v/s 43 asymptomatic AS	BNP higher in symptomatic patients	[19]
		Weber, 2006	102 undergoing AVR and 57 medically treated AS	NT-proBNP as independent prognostic information of adverse outcomes in medically treated patients	[20]
		Clavel, 2014	1953 patients with > moderate AS	Higher mortality with higher BNP levels	[22]
		Capoulade, 2014	211 asymptomatic AS	Higher BNP = Higher occurrence of adverse events	[23]
Troponin T	Reflects the muscular ischemic injury	Chin, 2014	131 patients	Torponin level associated with AVR or CV death	[24]
sST2	Reflects cardiovascular stress and fibrosis	Lancellotti, 2015	86 patients with AS	Independent predictor of cardiovascular event/heart failure	[26]
Galectin-3		Arangalage, 2016	583 patients with AS	No correlation between Galectin-3 and AS complications	[27]
Lipoprotein A (LpA)	Promotes atherosclerotoc stenosis and thrombosis	Arsenault, 2014	17553 participants	High level of a variant of LpA = increased risk of AS	[31]
		Kamstrup, 2014	77680 participants	Elevated LpA >90 md/dl = Increased risk of AS in the general population	[33]
Metalloproteases (MMP)	Inflammatory enzymes degrading collagen, elastin and proteoglycans in the matrix	Edep, 2000	9 AS patients v/s 4 without AS	MMP-1,-2 & −3 overexpressed in AS valves v/s normal leaflets MMP-9 is uniquely expressed in AS valves	[34]

However, these myocardial markers are not alone. Another group of biomarkers were reported as implicated in aortic stenosis, maybe directly involved in the calcific progression.

- Lipoprotein A (LpA) is a lipidic marker which has been reported to be the most correlated to the aortic calcification, as it reflects the evolution of the mineralization and calcification of aortic leaflets [[47], [48], [49]].
- As well, some of the Matrix Metalloproteinases (MMPs) were found over expressed in calcified aortic stenosis such as MMP-2, -3 and -9. They directly participate in the regulation of the extracellular matrix and could be involved in the early valvular remodeling [[50], [51], [52]].
- And many more publications reporting other potential biomarkers without sufficient evidence [18,[53], [54], [55], [56], [57]].

What if we have a look at more specific molecules, regulating their expression way before they are expressed in the tissues or blood?

## 5 Involvement of microRNAs (miRNAs) in aortic calcifications

In the same period of the macro-clinical evolution in the AS management, a micro molecule-related regulatory pathway started to get an increased importance in basic and clinical molecular research. Since miRNAs were first described in 1993 in *Caenorhabditis elegans*, clinicians and researchers have been involved in understanding their structure, action and expression in the tumoral cells and extending to other medical specialties, among them the cardiovascular field [58,59].

MiRNAs are small non-coding RNA sequences, of 21–23 nucleotides, regulating gene expression in mammalians, and modulating their RNA messenger translation in proteins. They can be directly transcribed in the nucleus from specific genes into precursor long pri-miRNAs latter cut by specific RNAses (canonical way) or can be secondary produced from the cleavage of introns or tRNAs, on special sites called ‘’mirtrons” (non-canonical way)’’. In both cases, the mature microRNA will create, with Argonaut proteins, the RISC complex which is destinated to interfere with its specific target mRNAs. This RISC complex can directly act on the mRNA inside the cell, or it can be carried, in the serum, to control other mRNAs in others cells [60,61]. This RISC complex bind the 3′UTR end of specific mRNA targets by base complementarity and control its protein expression [62].

MiRNA acts in two ways on its targets, either by destroying completely the target mRNA, decreasing then its seric concentration, or by blocking its translation.

MiRNAs are stable in the serum and can be easily measured by RT-qPCR to evaluate their expression in tissues or serum. These particularities confer the interesting role of biomarkers to the miRNAs [63].

As noticed earlier, miRNAs became routinely studied in the cardiovascular field in the late 2000's. With a more developed knowledge in the oncologic field, their action on different pathways of the cardiac disease has been widely published.

Atherosclerosis and aortic valve disease have been one of the subjects where miRNA found a well-established place and many researches are conducted to find a relationship between miRNAs and these fatal diseases [64]. Many studies address the role of miRNAs in several cardiovascular development [[65], [66], [67]], diseases [[68], [69], [70], [71]] but also therapeutics [64,72]. Many more studies and miRNAs are more widely used in cancer screening and follow-up, and even in cancer treatment. Our team published few years earlier a direct relation between miRNA and carotid atherosclerotic plaques [73,74] or cardiovascular events linked to chronic kidney disease [75,76].

The complex mechanism of aortic valve calcification implicates a huge number of molecules directly related to each step during the valvular structural modifications.

Studies concerning the miRNA implication in the aortic calcification started to be published in 2011. miR-26a, −30b and −195 were compared between stenotic and insufficient bicuspid valves and found to be under expressed in calcified aortic valves by decreasing the expression of Alkaline Phosphatase (ALKL), SMAD1, SMAD3, SAMD5, RUNX2 and BMP2, with their role in reprehending aortic calcifications. miR-195 increases the expression of BMP2, RUNX2 and SAMD 1,3,5 but increases also anticalcification genes such as JAG2 and SMAD7 [77]. In another study, miR-141 was under expressed in bicuspid valves when compared to tricuspid valves. This miRNA is a BMP2 regulator, by increasing its expression. BMP2 is directly implicated in the aortic valve calcification by regulating the osteogenesis [78]. The main BMP2 is also regulated by the miR-30b with a direct action on the apoptosis and the aortic calcification, as BMP-2 controls the osteoblastic differentiation by targeting RUNX2, SMAD1 and Caspase-3 [33]. miR-92a is also implicated in the angiogenesis and endothelial proliferation. Its inhibition improves the endothelialisation and decreases though the intimal lesion [79]. Our team found miR-92a significantly increased in bicuspid calcified aortic valves when compared to tricuspid valves, with a direct correlation with the severity of the aortic stenosis [80]. miR-148a-3p, expressed in the leaflets stretch and shear-stress, increases the expression of NF-κB signaling pathway with an increase of inflammation and then calcification [81]. The same shear-stress regulates the expression or miR-214, with its anti-calcification action which is under expressed in calcified valves when compared to normal valves [[82], [83], [84]]. miR-143 is over expressed in calcified aortic valves when compared to normal valves. It regulates directly the Matrix GLA Protein, implicated in osteogenesis [85].

In another publication, miR-122-5p, -625-5p et −30e-5p are decreased in stenotic valves while miR-21-5p et -221-3p are over expressed and both acting on the extracellular matrix [86]. miR-210 was reported as a prognostic and soluble marker of aortic stenosis according to a VO2 max measure [87]. Recently, in a recent study on bicuspid aortic valves, Poggio et al. found a difference in the endothelial cells reaction to oxidative stress, DNA damages and apoptosis via the p53 pathway, on a reduced pattern of calcified tricuspid and bicuspid valves (miR-26b-3p, -139-3p, -197-3p, -328-3p, −520g-3p, -561-3p, - 573 and -1180-3p) [88]. Finally, 4 miRNAs (miR-122, -130a, - 486 and −718) were reported as the seric signature of bicuspid aortic valves but, apart from miR-122 (see above) were not found in any other study [89] (Table 3).

**Table 3:** MicroRNAs implicated in the development of calcific aortic valve disease.

miR-	Action	Target	Expression	Valve Type	Reference
miR-92a	Anti-angiogenesis, favors endothelial proliferation		↑	Bicuspid	[53,54]
miR-26a miR-30b miR-195	↓ Calcification Apoptosis ↑ Calcification	ALKL, SMAD, RUNX2, BMP2 JAG - SMAD	↓ ↓ ↓	Bicuspid	[50,52]
miR-141	↑ Calcification	BMP-2	↓	Bicuspid	[[56], [57], [58]]
miR-148a-3p	↓ Inflammation in elongated lealflet	NF-κB	↓	Bicuspid	[[56], [57], [58]]
miR-214	↓ Calcification		↓		[[56], [57], [58]]
miR-143	Osteogenesis	MGP	↑	Calcified valves	[59]
miR-122-5p miR-30e-5p miR-625-5p miR-21-5p miR-221-3p	Extracellular Matrix, favors calcifications		↓ ↓ ↓ ↑ ↑	Calcified v/s normal valves Cadaveric Study	[60]
miR-26b-3p miR-139-3p miR-197-3p miR-328-3p miR-520-3p miR-561-3p miR-573 miR-1180-3p	Inadapted answer from the endothelial cells on oxidative shear stress Apoptosis through the p53 pathway. Inadapted reactions to DAN damage.	Gluthation Peroxydase (GPX3) Sulfiredoxin 1 (SRXN1)	↑ ↑ ↑ ↑ ↑ ↑ ↑ ↑	Bicuspid	[62]
miR-122 miR-130a miR-486 miR-718	Action on valvular calcification and aortic dilation in patients with bicuspid Aortic Valve, using the TGF-β pathway Vascular remodeling	TGF-β1	↓ ↑ ↓	Bicuspid	[61]

## 6 What are the perspectives related to these miRNA results?

**Fig. 2:**
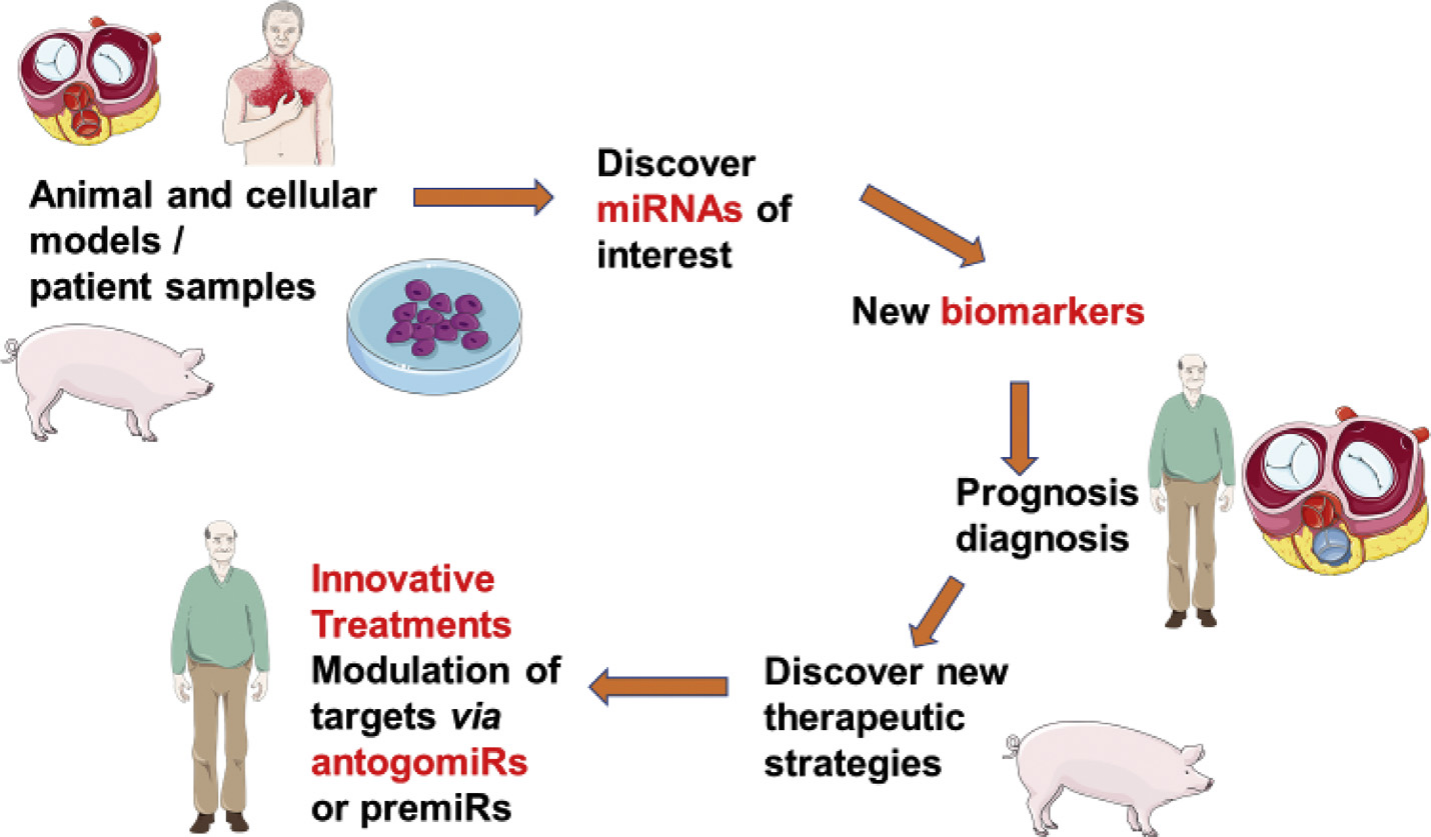
An outline to develop miRNAs as new biomarkers and innovative treatments in the field of valve calcification. Total RNA can be extracted from cell cultures, pre-clinical models. Up to a thousand miRNA expression can be titrated using either transcriptomics (eg. Microarray, next generation sequencing …) to detect the most deregulated miRNAs. These miRNAs can then be measured in patient cohorts to validate them as diagnostic or prognostic biomarkers. In long term up-of down-regulating their expression in selective tissues can be a new therapeutic approach.

The process from the isolation and identification of miRNAs in any disease is resumed in Fig. 2, with the different steps leading to the clinical application of miRNAs as diagnosis or prognosis biomarkers and evolving to the development of innovative treatment (Fig. 2). First of all, the direct relation between tissular and seric expression of miRNAs is an interesting hypothesis, leading to the use of miRNAs as specific biomarkers in aortic valve disease. This was demonstrated in other pathologies: for instance, in oral squamous cell carcinoma 30 out of 48 differentially expressed miRNAs were found to be deregulated in both tissue and serum of the same patients [90]. MiRNAs may reflect the importance of molecular evolution inside the valvular tissues by simply dosing their seric expression. In the case of aortic stenosis, miRNAs could help to diagnose patients with a higher risk of rapid calcification, such as patients with bicuspid aortic valves, in order to follow them more closely to propose the best suited treatment before the onset of ventricular complications of AS. Furthermore, patients undergoing a bioprosthetic replacement of their aortic valve may have their prosthesis choice modified in case they present a profile of rapid calcification. In this last case, a life-time mechanical prosthesis may be the best option to treat these patients.

On the other hand, the action of different miRNAs on different pathways of the valvular structural degeneration increases their interest in enhancing or inhibiting their role to act, directly on the main causes of the calcific evolution of the aortic valve. MiRNAs are, since many years, the target of ‘’antagomirs'’ and ‘’miR-mimics’’ [91,92], developed using molecular biology engineering. The objective of antagomirs is to downregulate the expression of specific microRNA leading to a reduced action of the targeted miRNA. At the opposite side, miR-mimics increase the expression of miRNAs enhancing though the expression and the action of other miRNA, upregulating the expression and the role of other pathways in aortic valve disease. These actions may prevent the onset of highly calcified aortic leaflet or just slow the evolution of the disease. These therapeutic actions are used in other diseases, especially in cancer screening, follow-up and treatment as seric biomarkers [93,94]. Encouraging results come from a very recent study from Toshima et al. in a pig model. They found that the expression of miR-34a, related to osteoporosis suppression and bone metastasis reduction due to an action on TGFβ-induced factor homeobox 2 [95,96], was enhanced in human calcified aortic valve, as well as *in vivo* and in vitro pre-clinical models. They evidenced in vitro, on isolated porcine Aortic Valve Interstitiel Cells (AVICs) that miR-34a plays a role in aortic valve calcification by modulating the Notch1-Runx2 pathway. They reproduced valvular endothelial damages in mice by scratching the leaflets with a wire introduced through the carotid artery. Last but not least, they showed that injecting locally or systematically an antagomir against miR-34a was able to significantly reduce the aortic valve calcification in their *in vivo* murine model. Their ground-breaking results suggest thus that miR-34a is a potential therapeutic target for calcific aortic valve stenosis, paving the way for human trials [97]. Specific tools are built in this very moment in the prospective of future clinical trials [98].

## 7 Conclusion

MiRNAs are expected to be the next generation of biomarkers in a large number of chronic diseases, including aortic valve calcifications. Targeting them to up- or down-regulate their expression is nowadays a challenge in molecular biology to interfere seriously with the molecular level of early modification to slower or treat the degeneration of native aortic valves. Several types of nucleic acids, including miRNAs are at the moment in phase II/III clinical trials and were proven able to modulate more than 20 targets in seven types of diseases [99]. Efforts are undergoing in the cardiovascular fields as was recently summarized. This clearly reflects the potential of nucleic acid therapies to treat intractable diseases and non-druggable targets.
